# Reflections on the Inclusive Co-Design Process of a Virtual Assistant for Individuals With Complex Care Needs: Mixed Methods Study

**DOI:** 10.2196/81941

**Published:** 2026-02-25

**Authors:** Julia F E van Calis, Kirsten E Bevelander, Anneke W C van der Cruijsen, Jenneken Naaldenberg, Geraline L Leusink

**Affiliations:** 1 Department of Primary and Community Care, Radboud University Medical Center, PO Box 9101, Nijmegen, 6500HB, The Netherlands, 31 24 3618181; 2 Academic Collaborative Intellectual Disability and Health - Sterker op Eigen Benen (SOEB), Nijmegen, The Netherlands

**Keywords:** mild intellectual disabilities, autism spectrum disorder, inclusive design, citizen science, inclusive research, sensitive virtual assistant

## Abstract

**Background:**

The digitalization of society has transformed daily life and health care, offering opportunities for accessibility and independence for individuals with complex care needs. However, users with limited digital skills still experience challenges because the technologies do not to align with their needs. Inclusive research and design approaches can improve technology by actively involving end users and stakeholders.

**Objective:**

This study investigated the experiences of co-researchers with a mild intellectual disability or autism spectrum disorder and other key stakeholders over time regarding the inclusive design process for a digital tool for individuals with complex care needs that was developed in a transdisciplinary consortium.

**Methods:**

The project that was examined applied an inclusive design process to develop a sensitive virtual assistant using the Vision in Product Design method and the design thinking approach. Nine consortium members, including 3 co-researchers, participated in semistructured interviews and a group discussion about the inclusive design process after each of the project’s 5 work packages (WPs). This resulted in 31 interviews and 5 group discussions in total. Individual experiences were gathered during interviews, and group discussions facilitated collective reflection. During the interviews, an adapted questionnaire was used for each WP with Likert scales and open-ended questions. The data analysis was conducted using a thematic approach and descriptive statistics for the questionnaire data.

**Results:**

Quantitative findings from questionnaires were complemented with qualitative insights from interviews and group discussions, with results presented chronologically per WP. The qualitative analysis resulted in 3 main themes: project approach, collaborative dynamics, and co-design in practice. Project approach showed how the team adapted its inclusive collaboration through expectation management, structured processes, and accessible materials. Collaborative dynamics described how communication and support evolved and how inclusive design principles were applied in practice. Co-design in practice outlined co-researcher involvement and content adaptations across the 5 WPs, highlighting how experiential knowledge directly informed design decisions. These findings show that inclusive collaboration developed over time and contributed meaningfully to both process and content.

**Conclusions:**

This study shows that, to accommodate an inclusive research and design process, tensions between project efficiency and meaningful inclusion need to be addressed, underlining the importance of continuous coordination, collaboration, and flexibility in transdisciplinary settings. Further, applying a stepwise approach in inclusive collaborations supports coordination, continuous evaluation, and flexibility. Inclusive methods, like preparatory activities, clear role division, accessible materials, and iterative feedback, enabled active co-researcher participation. These methods contributed to a shift in ownership, allowing co-researchers to gain greater influence and co-shape both the development process and the content. The findings provide insights into how to enhance equity and relevance in inclusive technology design for individuals with complex care needs, such as individuals with a mild intellectual disability or autism spectrum disorder.

## Introduction

The rapid digitalization of society has transformed the way in which individuals interact with technology in health care and in their daily lives [1,2]. As digital innovations become more integrated within society, it is essential that they meet the needs of diverse user groups such as those requiring complex care. These individuals often rely on a wide range of health and social support services, making accessibility to digital health tools particularly relevant. However, such tools can be difficult to use and less accessible for individuals with limited digital skills. For example, individuals with a mild intellectual disability (MID) or autism spectrum disorder (ASD) often experience difficulties acquiring digital literacy skills and using digital devices or the internet [3,4] because of limited cognitive skills and challenges with processing information efficiently [5]. Both groups frequently struggle with understanding complex instructions, problem-solving, and adapting to new technologies [5,6]. This can place them in a vulnerable position, as it limits their ability to fully participate in today’s complex society [7,8]. Digital tools designed to fit their living situation may reduce challenges, increase their empowerment, and contribute to more effective care, ultimately decreasing health disparities in these populations [9,10].

Although digital health tools can enhance accessibility and independence, they are often not developed to align with these users’ specific needs and skills [2,11,12]. Despite the growing inclusion of these target groups in technology research and design projects [11,13,14], the participation of end users with MID and ASD remains limited [15-17]. This gap highlights the need for inclusive research and design approaches that incorporate the perspectives of these end users in the development of health technologies [18-20]. In this study, inclusive design is defined as an approach to make the design and development process accessible, equitable, and participatory for individuals with diverse abilities and experiences. Drawing from the Vision in Product Design (ViP) methodology [21] and design thinking [22], inclusive design refers to a flexible, iterative process that values multiple forms of expertise, including experiential knowledge, and adapts its methods, materials, and facilitation strategies to ensure meaningful involvement of all participants.

User-centered approaches [23] place user groups at the center of the design process by using tailored methods to address their specific needs [24,25]. These approaches follow an iterative process, applying rapid prototyping and prioritizing end user experiences by collaborating within interdisciplinary teams [26]. Inclusive, flexible, and adaptive approaches have been shown to be well-suited to involving people without prior experience in technological design [11,14]. Recent work in inclusive participatory design further demonstrates how design processes can be adapted to respect diverse sensory, cognitive, and communicative modes of engagement [27-30]. These studies provide important methodological insights into how participation can evolve into equitable collaboration, ensuring accessibility and involvement across all stages of technological development. In research, inclusive approaches such as inclusive research or citizen science actively involve end users in all phases of technological research and development processes. In these approaches, end users are experts-by-experience and co-researchers [31,32] who serve as advisors, cooperators, or nonprofessional researchers alongside those with academic or design training to add value through the sharing of their lived experiences [13,33]. User-centered and inclusive research and design approaches can be applied to develop (health) care technologies that are not only functional but also accessible and usable for all [14,34]. However, no research has been conducted into how end users with limited digital skills experience collaboration in technological research and development processes or into the adaptations that are necessary to involve them.

In addition to the participation of co-researchers and experts-by-experience, the involvement of other stakeholders in technological research and development is essential [20,26]. Examples of these stakeholders include (informal) caregivers, support workers, IT developers, or other specialists to align with the end user context and support in practice [35]. This gives technologies a greater chance of successful implementation and use [18,36,37]. However, previous studies have found that less attention is given to involving key stakeholders and end users throughout all design iterations or to the continuous monitoring of the design process during implementation [20,26]. Reflecting on the collaboration during technology development and implementation phases can provide practical insights valuable for (future) development [26]. Therefore, this study aimed to examine how inclusive design methods facilitate the development of digital health tools, focusing on the experiences of co-researchers with MID or ASD and other stakeholders over time on the inclusive design process of a digital tool for individuals with complex care needs, developed within a transdisciplinary consortium. Rather than assessing the design outcomes of the digital tool itself, the research focused on the process-level learning, that is, the iterative adaptations, facilitation strategies, and collaborative dynamics that enabled or hindered inclusive participation over time. The research question guiding the analysis was: *What process modifications and content adjustments were made over time to accommodate an inclusive design process with co-researchers with MID or ASD in the development and implementation of a digital tool for individuals with complex care needs?*

## Methods

### Study Setting

Within this study, the inclusive design process for a digital tool in the form of a sensitive virtual assistant (SVA) for individuals with complex care needs was investigated. A consortium of transdisciplinary partners aimed to develop an SVA that could guide individuals with complex care needs toward appropriate support for questions related to mental and physical care [10]. Artificial intelligence technology was used to enable the SVA to adapt its responses in a sensitive manner.

The consortium consisted of 5 partners: (1) a university department with expertise in technology and chatbots in service settings; (2) an academic collaborative conducting research with and about people with intellectual disabilities; (3) a social design agency; (4) a foundation for social care organization informing, advising, supporting, and employing people with complex care needs such as individuals with ASD and intellectual disability; and (5) an IT company. In this project, 3 individuals with ASD or MID, representing the target group, participated as co-researchers and experts-by-experience throughout the entire research and design process. The co-researchers work for the academic collaborative and the social care organization. Their involvement enabled the consortium to consider the challenges underlying the complexity of different living and care situations. Their roles varied across project phases such as sharing lived experiences, co-designing prototypes, recruiting participants, co-moderating focus groups, and preparing data collection materials. By combining inclusive research and user-centered design, the project aimed to ensure meaningful participation.

**Figure 1.**
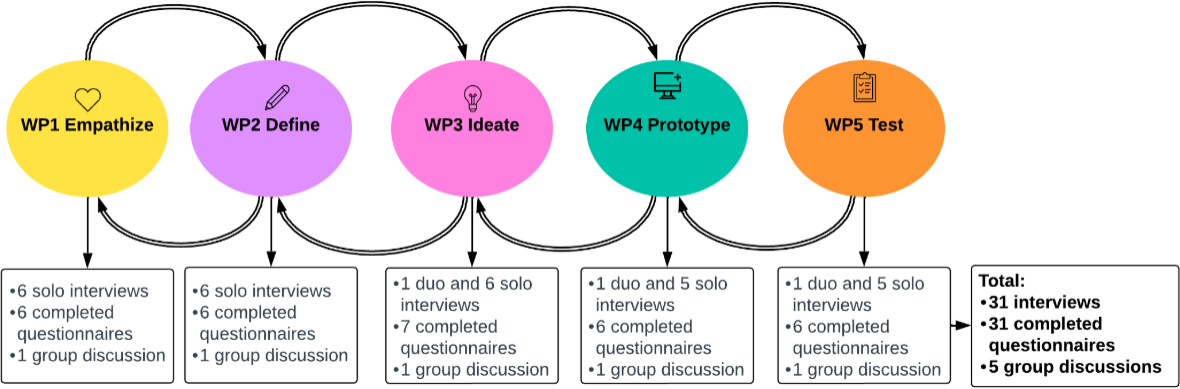
Flowchart of the sensitive virtual assistant (SVA) project and data collection for the reflection on the inclusive design approach.

The SVA was developed using the ViP method [21], incorporating the design thinking approach [22]. Each design thinking phase was operationalized as a separate work package (WP1-WP5), aligned with the steps of the ViP method (Figure 1) [22]. In the Empathize phase (WP1), the focus was on identifying and mapping the needs of the end users through focus groups and a literature review. The Define phase (WP2) analyzed the insights from WP1 to develop a user profile, which included user needs and desired experiences. In the Ideate phase (WP3), a wide range of creative solutions was generated. Among these activities, personas were created, representing fictional user profiles designed to represent typical users’ behaviors, motivations, and needs. Additionally, the development of user scenarios and user journeys helped define functional requirements and typical user experiences. In the Prototype phase (WP4), these ideas were turned into early-stage functional designs (ie, low-fidelity prototypes) and tested for usability and performance. This phase also included making the ethical approval application for user testing. Finally, the Test phase (WP5) involved developing 3 functional versions of the SVA, each with a different interaction style. These were tested with real users in actual care settings to evaluate and refine the solution.

### Study Design

This study used a mixed methods approach to reflect iteratively with co-researchers and consortium members on the SVA’s inclusive design process over time; see Figure 1. The SVA functioned as a context and illustrative case that allowed the research team to analyze inclusive processes in practice rather than to evaluate the final design outcome. Qualitative data were gathered through (1) semistructured interviews with co-researchers and key stakeholders followed by (2) group discussions with co-researchers at the end of the 5 phases or respective WPs. During the group discussions, the co-researchers reflected together on the insights from the interviews. Quantitative data were collected during the semistructured interviews by filling in a digital questionnaire adapted for each WP. The integration of qualitative and quantitative methods ensured structural reflection over time across the entire process with all participants. Further, this study was conducted in collaboration with a co-researcher with an MID, whose perspective was actively integrated into the research process.

### Participants

Representatives from all 5 consortium partners participated in the study, with a total of 9 participants, including the 3 co-researchers. The other 6 participants were consortium partners involved in various roles: researchers, social designers, project leader, and technology developers. Table 1 shows the participants’ distribution in the interviews and questionnaires. The first 3 group discussions took place with 3 co-researchers, and the last 2 discussions took place with 2 co-researchers.

**Table 1.** Participant distribution in the interviews.

Total participants per work package (WP)		Co-researchers, n	Researchers, n	Social designers, n	Project leader, n	Technology developers, n
WP1 (n=6)		3	1	1	1	0
WP2 (n=6)		3	1	1	1	0
WP3 (n=8)		3	1	1	1	2
WP4 (n=7)		2	1	1	1	2
WP5 (n=7)		2	1	2	1	1

### Ethical Considerations

Primary data collection was conducted in compliance with the current data protection regulations of the General Data Protection Regulation and the Declaration of Helsinki. Each participant received an information letter and provided informed consent via a comprehensible consent form. Both documents were co-created with the co-researcher with MID to enhance accessibility (eg, by incorporating simple text, graphic support, large font, and increased white space). Data collection was anonymous, and no conclusions can be drawn about individuals in the presentation of results. Since the participants were part of the consortium and were compensated for their input as members, they were not compensated separately for this study. The entire study was submitted for ethical review to the Research Ethics Committee CMO Radboudumc and waived from further ethical assessment (reference number 2023‐16400) because it does not constitute medical scientific research according to the Medical Research Involving Human Subjects Act (WMO) [38]. This waiver is consistent with national and institutional policies that specify that research falling outside the scope of the WMO does not require further review. Relevant guidance can be found in the CCMO framework for non‑WMO research (CCMO, *Niet‑WMO‑onderzoek*)[39], as well as the CMO Radboudumc policy on non‑WMO assessment[40].

### Procedure and Data Collection

The aim of this study was to identify key adaptations in both the collaborative process and the content of inclusive research practices over time (May 2023–May 2024). After each WP was completed, semistructured interviews were conducted with the consortium members, including the co-researchers. These semistructured interviews focused on participants’ engagement, lessons learned, and overall experiences and were conducted with 6 to 8 participants per WP. As the interviews were conducted after each phase, experiences were still recent and therefore easier to retrieve. During the interviews, quantitative data were collected using digital questionnaires adapted for each WP. The interviewer filled in the quantitative responses using *I Co-research* (*IkOnderzoekMee*; Crowdience), an inclusive digital research platform for accessible dissemination of questionnaires [14,41]. The questionnaires consisted of quantitative elements using Likert-scale items (see Table 2), incorporating simplified text, graphic support, large fonts, and increased white space to facilitate the participants [14]. Examples of how the scales were used in the questionnaires can be found in Multimedia Appendix 1. During the interviews, open-ended questions were asked regarding specific activities of the WP and the clarity of roles and tasks within these activities, experiences within the collaboration in the consortium, goals of the WPs, lessons learned, and individual reflections. The open-ended questions provided a deeper understanding of how adaptations were implemented and experienced. Multimedia Appendix 1 provides the interview guide combining the interview questions and a WP1 questionnaire. Following the interviews with questionnaires, a group discussion was held with the 3 co-researchers involved in the project. During these discussions, insights, and areas of interest from the interviews and questionnaires such as participant engagement, challenges, and overall perceptions were discussed. An independent researcher (JFEC) moderated these sessions. In line with the paradigm “nothing about us without us” of the international treaty from the United Nations Convention on the Rights of Persons with Disabilities [42], the data collection process was designed to ensure that the voices of co-researchers with MID or ASD were central. At the same time, consortium members without disabilities were included to investigate how inclusive collaboration was experienced and facilitated across roles. This comprehensive inclusion allowed us to identify how the participatory process was shaped. Importantly, although all perspectives were collected, the reflections of co-researchers in the group discussions formed the primary reference point for interpreting and discussing results in later stages of the analysis.

**Table 2.** Scales used in the questionnaire.

Type of scale	Color	Points	Distribution (lowest to highest)
Visual analog scale	Black and white	10	1=Totally not clear, 10=Very clear; 1=Totally not nice, 10=Very nice
Stars	Yellow	5	1=Very negative, 5=Very positive; 1=Little contribution, 5=Great contribution
Smileys	Green, yellow, orange, and red	4	1=Very good, 4=Bad
Thumbs	Green, yellow, and red	3	1=Yes, 2=Partially, 3=No

#### Data Analysis

Descriptive statistics were applied to analyze the quantitative data from the digital questionnaires. Microsoft Office Excel was used to calculate frequencies, median scores, and range distributions, allowing for a clear overview of participants’ response patterns, medians, and range distributions.

All interviews and group discussions were recorded and transcribed for analysis. Table 3 presents the consecutive steps of the qualitative data analysis, which was conducted in multiple phases using a thematic approach. After each WP, the transcripts of the semistructured interviews were rapidly coded [43,44] to gain an initial impression of participant engagement, challenges, and overall perceptions. The results from this phase were included in the subsequent group discussions with the co-researchers. During these discussions, key themes based on the research questions were identified, forming an initial framework to guide the analysis of the semistructured interview data of the corresponding WP. This framework guided further rapid coding of the interview and discussion data to identify overarching themes and subthemes [43-45]. The analysis process was conducted separately for each WP, ensuring a structured and iterative evaluation of the data.

In addition to the thematic analysis of interview and group discussion data, a structured analysis of co-researcher involvement and activities per design phase was conducted (Table 3, step 8). This analysis, based on data extracted from the interviews, focus groups, and a logbook, resulted in a table summarizing the activities conducted within each WP, the content adaptations made, and the involvement of co-researchers in these activities, supported by participants’ experiences.

**Table 3.** Consecutive steps, actions, and aims of the coding process.

Step	Action	Aim
1	Reading transcripts of interviews	Become familiar with data and sections of the transcripts
2	Initial rapid coding (JFEC, AWCC) of the semistructured interviews and questionnaire responses after each work package (WP)	Gain first impression of interview data and explore areas of interest such as participant engagement, challenges, and overall perceptions
3	Using first impression on which to reflect in the subsequent group discussions with the 3 involved co-researchers (JFEC, AWCC)	Identify key themes and reflections based on research question and create initial coding framework with thematic categories
4	Applying initial coding framework and using rapid coding approach to guide second analysis	Conduct bottom-up coding, identify overlap and connections between codes, and ensure collective reflection
5	Clustering of codes into broader themes relating to the research question (JFEC, AWCC, KEB)	Identify emerging and overarching themes and subthemes
6	Discussing clusters of codes using insights from group discussions (JFEC, AWCC, KEB, JN)	Refine subthemes and analyze their relationships within each main theme, creating the final coding structure
7	Repeating steps 1-6 for each WP separately	Generate an overview of insights per WP
8	Collectively analyzing WPs	Develop a comprehensive overview of the full process and conduct qualitative data analysis, which resulted in 3 main themes: project approach, collaborative dynamics, and co-design in practice

## Results

### Themes

**Figure 2.**
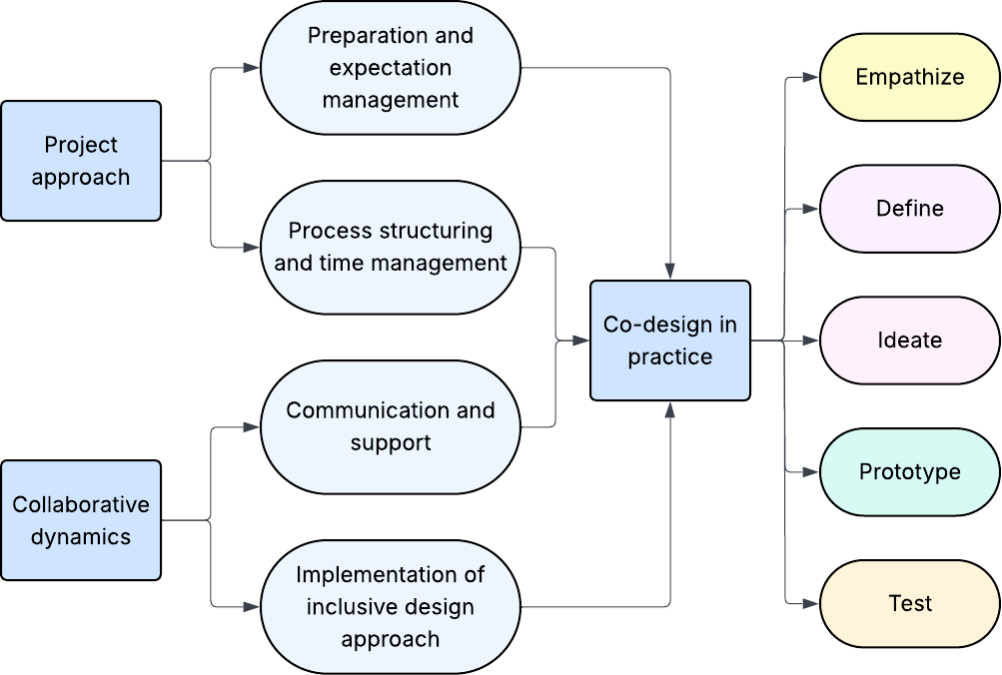
Thematic coding map of the inclusive design process.

The thematic analysis resulted in 3 main themes that together describe how inclusive collaboration was shaped and experienced throughout the design process. The thematic coding map of the inclusive design process is visualized in Figure 2. The first 2 themes show how the team adapted methods, facilitation, and collaboration over time. The subthemes are first represented by quantitative results from the questionnaires then further illustrated by qualitative results with quotes from the interviews and group discussions. The last theme gives an overview of the core activities, co-researcher involvement, and content adaptations to the inclusive research and design process during each WP illustrated by participants’ quotes about experiences. The results are described chronologically structured by the 5 WPs. Because the SVA functioned as a use case, the emphasis here is on process adaptations rather than design evaluation of the tool itself. Where relevant, the results indicate whether adaptations were particularly beneficial for the co-researchers with MID or ASD or more generally valuable for all consortium members.

All quantitative outcomes have been integrated into a single comprehensive table. Table 4 presents these results collectively, offering a consolidated overview of the rating ranges and median scores across all WPs.

**Table 4.** Quantitative outcomes of the grading of all work packages (WPs).

Grading for	Scale	WP1 grade (n=6)		WP2 grade (n=6)		WP3 grade (n=7)		WP4 grade (n=6)		WP5 grade (n=6)	
Range	Median	Range	Median	Range	Median	Range	Median	Range	Median
Contributions from the co-researchers to achieving the goals of the WP	1‐5 stars (Low to High)	2‐3	3	3‐4	3	3‐4	3.5	2‐5	4	3‐5	4.5
Clarity of tasks within each WP	Visual analog scale 1‐10 (Low to High)	5‐8	7.5	2‐10	6.5	2‐8	7	3‐10	7	5‐9	7.5
Overall experience of the collaboration within each WP	Visual analog scale 1‐10 (Low to High)	3‐5	4	3‐5	4	3‐5	4	3‐5	4	3‐5	4.5

### Project Approach

Three subthemes describe how the team adapted the project approach to facilitate inclusive collaboration: preparation and expectation management, process structuring and time management, and the accessibility and clarity of materials and meetings.

#### Preparation and Expectation Management

Table 4 shows the grading for the co-researchers’ contributions to achieving WP’s goals. The median ratings went from low to medium to high across the WPs, with a widening grade range particularly in WP4, suggesting both improvements as well as ongoing difficulties in the contributions in achieving the goals of each WP.

In WP1, considerable time was spent preparing the project and designing the research approach. This led to a strong methodological foundation by combining the ViP approach with clearly defined research questions aimed at developing the SVA in a grounded and well-substantiated way. This preparatory phase was experienced as valuable by the participants: “A lot of work before the start, especially substantiating the fundamental research. That intensive pre-application phase is paying off now*”* [Group discussion WP1, co-researcher 1]. Although these efforts were experienced as beneficial, participants emphasized that role clarity and mutual expectations were not sufficiently addressed in this phase. Co-researchers noted that roles and meeting purposes were not always clear, thereby hindering inclusive participation: “The division of roles and their descriptions should be clearer in advance, and the purpose behind a meeting should also be better explained” [Group discussion WP1, co-researcher 3].

In WP2, the co-researchers’ role was more clearly defined, and preparatory work was focused on aligning tasks with their expertise and ensuring that they were adequately informed to contribute meaningfully to the research activities. The participants stated that, in WP3, issues identified in WP1 and WP2 were addressed, such as more structure and explicit planning, leading to process improvements. In WP4, expectations were more explicitly defined, as indicated by co-researchers. Preparations for WP5 were also experienced as well-structured. However, uncertainty about project progress remained a recurring point, especially regarding coordination with external partners. Despite the high workload and challenges, WP5 was experienced as successfully completed. Nevertheless, reflections on the full project indicated that expectation management on roles and expectations should have been addressed earlier. Clarifying roles and meeting purposes proved beneficial for all team members, while the clearer explanation of individual tasks and meeting goals was particularly important for the co-researchers to understand their expected contribution.

#### Process Structuring and Time Management

Process structuring and time management varied across the WPs. In WP1, a co-researcher was present at all focus groups acting as a second chairperson, contributing to continuity. In WP2, the team revisited earlier steps when clarification was needed and repeated certain elements of the methodology; this was experienced as supportive and necessary: “More attention was paid to repeating and explaining the steps, so planning those extra sessions was really helpful” [Interview WP2, consortium member 1]. These repetitions and slower pacing were adaptations that specifically supported the co-researchers by providing predictability and reinforcing understanding, while improved scheduling and process visibility benefited the entire consortium.

Co-researchers reported that certain steps in the process had been skipped or insufficiently addressed. They felt they had not been adequately informed, which led to uncertainty about their role in specific sessions. This was especially apparent in WP2, where feedback loops discussing and revisiting important aspects of the care setting were not applied consistently over time, leading to confusion, a feeling of unpreparedness, and missing structure among some co-researchers: “I didn’t get enough explanation about what was expected of me, so it felt like some steps were missing and I wasn’t well prepared. It felt like the structure was missing a bit” [Interview WP2, co-researcher 3]. In WP3, the design process was structured using personas and value scenarios. These tools helped visualize user needs and supported decision-making. The use of visual tools was initially introduced to make abstract information more accessible for the co-researchers but later proved equally valuable for all team members to align perspectives and trace decisions. However, not all choices made during the design process were clearly traceable to earlier input, consequently raising questions about the origin of decisions: “It’s still unclear where some decisions and information come from, but I do have confidence in the process and what it will lead to” [Group discussion WP3, co-researcher 2]. In WP5, the fast pace of data collection limited opportunities for deeper engagement, particularly among co-researchers not involved in day-to-day activities. This moderate level of involvement indicates the tension between project deadlines and inclusive participation.

#### Accessibility of Meetings and Clarity of Materials

The low to high gradings indicated varying experiences with the clarity of the tasks within each WP (Table 4). These variations are reflected in both improvements and persistent challenges in the clarity of materials and meetings.

Throughout the project, accessibility of documents and the clarity of meeting structures were important for structuring inclusive collaboration. In WP1, the importance of aligning language and terminology across disciplines was raised. One co-researcher explained how this led to misunderstandings: “There was a moment I got stuck on the terminology, some explanations were missing, so I misinterpreted terms like ‘advisory group’ and focus group’” [Interview WP1, co-researcher 1].

In WP2, the team experienced difficulties with access to shared documentation because of unclear file structures and inconsistent uploading of materials. Despite this, valuable insights were still generated through collaborative analysis sessions. In addition, some participants found the abstract language used by the social designer difficult to follow, particularly during the initial explanation of the design concepts and framework. This suggests that the issue was not limited to participants with MID or ASD. Sessions about the design requirements were considered too complex or intensive, thereby negatively impacting participation. There was a clear need for better guidance and coordination: “That session was much harder than I expected. I underestimated the approach, reading all the design requirements aloud was too much for 1 co-researcher, especially with all 3 co-researchers in 1 session. Better preparation would have helped” [Interview WP2, consortium member 3]. Simplifying terminology and using concrete examples primarily supported the co-researchers, whereas improvements in file organization, consistent upload practices, and meeting agendas were generally beneficial for all participants.

During WP3, difficulties in managing large amounts of information were expressed, particularly regarding working with personas. This emphasized the need for better preparation and briefing. Collaboration in smaller groups improved clarity, and strategic selection of who attended meetings helped structure discussions more effectively: “It was helpful that we could build on earlier experiences and realized that sometimes it’s better to meet in smaller groups and have a clear agenda with a focused question for the co-researchers” [Interview WP3, consortium member 4]. This adaptation was initially designed to meet the communication preferences of the co-researchers but was later appreciated by the entire team for improving efficiency and focus. In WP4, the inconsistent involvement of co-researchers in different sessions revealed the need for a more transparent selection and planning process; this was subsequently addressed by clarifying the tasks and communicating planning and agendas more openly and in advance.

### Collaborative Dynamics

This theme describes how the team experienced and shaped collaboration throughout the project. We identified 2 subthemes: (1) communication and support and (2) the implementation of the inclusive design approach.

#### Communication and Support

Over the course of the project, the importance of structured communication and tailored support became more evident, with improvements throughout all phases. In WP1, the involvement of a researcher with experience working with the target group contributed significantly to inclusive interaction and understanding. This early investment in a collaborative dynamic created a foundation that contributed to more effective coordination and inclusive dialogue in later phases, although some team members indicated that communication within the broader group was occasionally fragmented in WP2 and WP3. The team became more aware of the need for regular status updates and accessible coordination, particularly for co-researchers. These structured updates were introduced to help co-researchers stay connected and informed. In WP3, this learning resulted in more targeted communication and the introduction of structured feedback sessions. One co-researcher reflected: “I sometimes miss status updates, and, as a result, I feel less connected to the group and the project” [Group discussion WP3, co-researcher 3].

From WP4 onwards, lessons from earlier phases were applied. Project roles became more clearly defined, and coordination across team members and with co-researchers improved. For example, responsibilities for preparing test sessions were allocated in advance, and co-researchers were actively involved in reviewing prototypes, leading to smoother collaboration and more targeted feedback. In WP5, external factors such as delays in receiving feedback from the tests posed challenges, partly because of scheduling difficulties and the time required for internal approvals. The team members emphasized the value of having expertise with inclusive approaches embedded within the team from the start: “Looking back, I now see how important it is to have knowledge and experience with the target group. This must be secured in advance” [Interview WP5, consortium member 2]. Overall, the experiences with communication and support illustrate a learning trajectory in which the team increasingly recognized and applied the conditions required for sustainable inclusive collaboration. The growing emphasis on inclusive communication was therefore both a targeted accessibility measure for the co-researchers and a general facilitator of cohesive teamwork.

#### Implementation of an Inclusive Design Approach

Consistent gradings were present for the overall experience of the collaboration within each WP (Table 4), indicating that this was experienced as fairly stable.

In WP1, the team paid attention to aligning tasks with co-researchers’ strengths and availability. These early decisions, such as involving co-researchers in defining project goals and choosing methods, helped create a foundation for shared ownership and equitable participation. This was reflected in how co-researchers took the initiative during planning meetings and felt empowered to provide critical feedback on design choices. In WP2, several team members and co-researchers described stronger group dynamics and increased peer support during meetings, suggesting a growing sense of cohesion. The team became more responsive to individual needs and made deliberate choices about when and how to involve co-researchers. This thoughtful approach contributed to a respectful and adaptive working environment: “There is a conscious decision whether or not to involve specific consortium members or co-researchers in certain activities. Their added value or potential overload is always considered carefully” [Group discussion WP2, co-researcher 2]. Adjusting task complexity and workload was particularly beneficial for the co-researchers, supporting autonomy and confidence in their role.

In WP3, the inclusive approach was further strengthened. Co-researchers suggested setting up a test group; this marked a shift toward more proactive engagement. Their suggestion was adopted and built upon in WP4 and WP5, where co-researchers played active roles in testing and presenting outcomes to various stakeholders, including care and support organizations, technology developers and intended target users. These contributions were experienced as motivating and valuable: “For me, it was a real highlight that I got to join and answer questions at an event. It made me feel even more part of the team” [Interview WP5, co-researcher 1]. Throughout the project, the team also gained insight into how different forms of experiential input could be used effectively. The distinction between experiential expertise (derived from personal lived experience) and experiential knowledge (the ability to reflect on and articulate that experience for others) became more visible, leading to more intentional use of individual contributions. For example, during user testing phases, some co-researchers contributed best by evaluating content based on their personal experience, whereas others translated those insights into concrete design suggestions. This initiative specifically empowered the co-researchers, reinforcing their agency within the design process while also fostering a stronger sense of shared ownership of the team.

### Co-Design in Practice

A summary of the core activities within the design phases, co-researcher involvement, and content adaptations to the inclusive design process made across the 5 different WPs is provided in Table 5. The adaptations were informed by the input from and collaboration with co-researchers. The following section elaborates on the rationale behind these adaptations resulting from this input, and reflects on participants’ experiences with the content adaptations made.

**Table 5.** Summary of core activities, co-researcher involvement levels, and content adaptations.

Phase, core activities, and level of co-researcher involvement	Description of co-researcher involvement	Content adaptations to inclusive research practices
Empathize (map the end users’ needs): conducted focus groups to gather insights from end users and performed a literature review to identify user challenges and context		
High	Co-researchers were actively engaged throughout all phases of the focus group (preparation, recruitment, data collection, and evaluation).	Themes for the focus groups were refined collaboratively with co-researchers to ensure inclusiveness by representing diverse care perspectives.
Limited	In the literature review, co-researchers were kept informed, contributed to discussing the research question, and reviewed summaries.	The literature review content was contextualized using insights raised by co-researchers during earlier discussions.
Define: (analyze insights from Empathize phase to create a user profile of the intended end users with the needs and desired experiences): identified and clustered contextual factors into core care intentions and developed an interest framework to capture users’ underlying motivations		
Low	Co-researchers contributed to reviewing the contextual factors but were not involved in clustering them.	Terminology and categories were adjusted in response to co-researcher feedback to improve clarity and accessibility for all consortium members.
Moderate	Reasonably active contributions in developing the interest framework were supported through peer conversations and guided sessions.	Interest profiles (summaries of key user motivations) and design missions (goals guiding the design process) were co-developed with co-researchers and refined based on their feedback to ensure alignment with users’ real needs.
Ideate (generate a wide range of creative solutions): created user scenarios based on complex care experiences, mapped user journeys and defined functional needs and designed personas (fictional profiles representing typical users), and formed test groups to explore concepts collaboratively		
High	The co-researchers co-created scenarios.	Scenarios were modified to reflect lived experiences and concrete examples provided by co-researchers.
Moderate	Feedback was provided by the co-researchers during sessions on the mapped journey and functional requirements.	Personas and requirements were refined based on feedback from the co-researchers to ensure alignment with real-world user experiences. Tools were used to visualize the input of the co-researchers.
High	Test groups were formed and initiated by a co-researcher.	Recruitment effort via the consortium network resulted in diverse groups, including individuals with lived experience and professionals.
Prototype (create functional designs and explore them through performance and usability tests): created and tested low-fidelity prototypes (simplified early versions) of the sensitive virtual assistant and prepared and submitted an ethical approval application for user testing		
High	The co-researchers actively interpreted and responded to user feedback.	Prototype elements, such as navigation, instructions, and tone of voice, were adapted by the co-researchers during user feedback sessions.
Moderate	A co-researcher supported the drafting of the ethical application to test the prototypes.	Ethical approval documents, such as the information and informed consent letter, were edited in consultation with a co-researcher.
Test (evaluate functional designs with real users to refine and improve the solution): developed 3 functional sensitive virtual assistant versions with different interaction styles and evaluated these versions in real care environments with end users		
Moderate	Co-researchers contributed insights during evaluation sessions, which informed improvements to the sensitive virtual assistant.	Adjustments to interaction styles (eg, tone, responsiveness) in the 3 tool versions were explicitly informed by earlier co-researcher feedback regarding communication preferences.
Moderate	Co-researchers played a key role in guiding the evaluation, interpreting user feedback, and expressing a strong sense of ownership in the process.	Adjustments were made to enhance usability, clarity of instructions, and emotional accessibility based on real-world feedback from participants and co-researchers.

In WP1, the focus group themes and literature review content were collaboratively refined with co-researchers, enabling diverse care perspectives to shape the discussions and ensure inclusive representation and contextual relevance. One consortium member reflected that they unintentionally excluded co-researchers from the later stages of the literature review, as they still saw it as a task that they had to carry out individually: “I did discuss with the co-researchers what would be a relevant research question, but after that, I unconsciously no longer really involved them because I still had the idea that this was something I had to do myself*”* [Interview WP1, consortium member 1].

WP2 showed that structured feedback rounds were used to discuss and cluster contextual factors. The process and individual roles were initially unclear to some co-researchers, which led to adaptations to improve task explanation and structure for greater accessibility: “My task was not explained well. This made it unclear what my specific contribution was. We discussed this, and it was clearer afterwards*”* [Interview WP2, co-researcher 2]. The iterative co-development of an interest framework was supported by facilitation from the researcher; these sessions involved a process of adaptations: “It was very helpful that the postdoc supported the translation of the interest framework so that it could be properly presented, even though it took some trial and error*”* [Interview WP2, consortium member 3]. Terminology, categories, interest frameworks, and design missions were adapted based on co-researcher input to enhance clarity and alignment with user perspectives.

The scenarios and personas were informed by the co-researchers’ concrete input; their contributions reflected real-life situations and ensured the inclusion of diverse user experiences. Tools, like an interactive online board where team members could brainstorm and collaborate, enhanced clarity and enabled their lived experiences to be visibly integrated: “There was a strong sense of contribution; you can see that on the board with all the input we have generated*”* [Group discussion WP3, co-researcher 3]. One co-researcher illustrated the challenges, caused by strict ethical requirements, regarding the planning of test groups: “I found setting up this plan challenging, especially because of the strict requirements of the ethics committee*”* [Interview WP3, co-researcher 1].

In WP4, prototype elements and ethical documents were co-developed with co-researchers to support usability, comprehensibility, and accessible communication. Co-researchers’ clear understanding of their role, as exemplified during feedback sessions, appeared to support their engagement and involvement in the testing process, as suggested by their proactive participation and comments on ownership: “When testing, I knew exactly what to do, that worked very well*”* [Group discussion WP3, co-researcher 2]. However, ethical procedures caused frustrations among the team, with 1 co-researcher describing his frustrations: “Meeting all these requirements was time-consuming and sometimes frustrating*”* [Interview WP4, co-researcher 1].

In WP5, interaction styles and instructional clarity were refined based on participant and co-researcher feedback to improve responsiveness, accessibility, and emotional engagement. The initial design of the functional tool versions was shaped more by prior input than by direct co-creation in this phase, contributing to stress and frustration when delays in deployment occurred: “I really had no idea if it would work; that was stressful” [Interview WP5, co-researcher 1]. Despite this, the testing phase was positively evaluated because of adequate preparation: “My tasks during the testing were clear because the postdoc had provided good explanations in advance*”* [Interview WP5, co-researcher 2].

Together, the adaptations and accompanying reflections demonstrate that co-researcher involvement shaped not only the content but also the form and accessibility of the research process. They highlight how continuous feedback and flexible collaboration were essential to aligning the design with real-world needs. Overall, accessibility-focused adaptations such as simplified language, smaller group sessions, and pacing adjustments were particularly supportive of the co-researchers, whereas measures like role clarification, structured planning, and consistent documentation were more generally beneficial for all consortium members.

## Discussion

### General Findings

This study reflects on an inclusive and iterative design approach involving co-researchers with MID or ASD in the development of a digital health tool, examining how inclusive design methods facilitated the development of the tool and shaped collaboration within the consortium. Through adaptations, such as thorough preparation, role clarity, accessible communication, and iterative feedback, co-researchers made meaningful contributions to the inclusive research and design process. Three overarching themes, project approach, collaborative dynamics, and co-design in practice, provided insights into how inclusive collaboration was accommodated throughout different design phases. The inclusive collaboration was supported by structured engagement, cognitive accessibility strategies, and iterative methods, which collectively facilitated a greater sense of ownership and active involvement over time.

This study showed that the use of a flexible, inclusive framework deepened stakeholder engagement and enabled concrete content adaptations through its stepwise approach, in this case through design thinking [22,46]. However, design thinking is not the only framework suitable for inclusive development. Inclusive design frameworks such as design for the whole population by Clarkson et al [47], universal design by Goldsmith [48], and user-sensitive inclusive design by Newell et al [25], offer complementary approaches that emphasize accessibility, user diversity, and context-sensitive solutions. In addition, complementary frameworks such as the CeHRes (Center for eHealth Research) Roadmap [26] and the NASSS (Non-adoption, Abandonment, Scale-up, Spread, and Sustainability) framework [49] are other valuable approaches emphasizing stakeholder alignment and long-term implementation contexts, respectively. The ViP method used to develop the digital tool draws from these traditions and emphasizes collaborative approaches, shared ownership, and flexibility across project phases [21]. Involving co-researchers in the design process made it possible for them to actively influence both the collaborative workflow and the development of the tool itself. This participatory approach not only shaped the structure of the design meetings and the inclusivity of the design materials but also enhanced the value and the applicability of the outcomes [32,50]. This study contributes experiential and theoretical insights into how inclusive design methods can be operationalized within transdisciplinary (health care) technology development.

Within the flexible and iterative approach, the tension between project efficiency and meaningful inclusion emerged as an ongoing challenge, as it required increased time, support, and adaptive capacity from the team. This tension has also been shown in previous studies, particularly in the context of complex design environments where timelines are tight and deliverables fixed [51,52]. Future research could therefore investigate how inclusive design practices affect collaborative dynamics and decision-making processes in project environments such as planning or iterative technology development. This would help clarify how to balance inclusivity with time constraints and resource limitations and offer practical strategies for managing this tension [31,51]. Understanding the value of shared expectations [53] and how teams navigate this can inform inclusive design processes that aim for meaningful inclusion throughout all phases of research and design [32].

Further, a shift toward co-researcher ownership emerged in the collaboration. In the earlier phases of the project, co-researchers were involved primarily in predefined tasks, whereas, in later phases, they initiated concrete activities such as forming user test groups. This illustrates how lived experience can transition from consulting to initiating in the design process [54]. The active involvement of co-researchers enhanced both the usability and the real-world relevance of the tool. This finding aligns with previous research suggesting that long-term, repeated engagement with end users allows them to influence the design process more deeply and ensures outcomes that better reflect their needs [50,55]. Cognitive accessibility, through visual aids, simplified materials, repetition, and small group formats, was particularly important in early phases and supported the meaningful contributions from the co-researchers [56,57]. This relates to prior literature on accessible formats [52] and dedicated support time [56] in inclusive research and technology development. Future research could further explore how to embed these inclusive practices in technology development projects with interdisciplinary teams and the involvement of co-researchers while maintaining methodological integrity and meaningful participant roles [13,31,32].

### Strengths and Limitations

A key strength of this study is its design, which included structured reflection moments after each of the 5 design phases. This approach enabled the participants to share their experiences immediately after each phase while also identifying changes and adaptations across the full design trajectory. Another strength lies in the mixed methods approach, which combined qualitative interviews and group discussions with tailored quantitative questionnaires executed within an inclusive digital platform [14]. Using both experiential insights and continuous group reflections allowed the team to adapt the collaboration dynamically. As a result, the co-researchers could engage more meaningfully, which led to deeper, more context-specific findings [54]. Some limitations must be acknowledged. The study was conducted within a single consortium, working on 1 digital (health) tool in a specific context. Additionally, the relatively small sample size may limit the diversity of perspectives captured and the broader applicability of the results [58]. Therefore, future research should examine other and larger settings.

### Recommendations

Drawing from the findings of this study, a set of practical recommendations for researchers and designers aiming to conduct inclusive design with individuals with complex care needs, such as individuals with MID or ASD [59,60], was formulated. These recommendations translate the experiential insights of the inclusive design process into actionable guidance for future research and practice:

- *Engage end users early and continuously*. Involve individuals with MID or ASD from the start of the project and maintain their involvement across all design phases. Early participation fosters ownership, while continuous engagement ensures that adaptations remain relevant as the project evolves.
- *Adapt sessions to fit participants’ abilities and needs*. Prepare accessible materials and ensure that meetings are structured, predictable, and easy to follow. Use clear and jargon-free language, concrete examples, and visual supports. Naming, visuals, and text should be recognizable and understandable.
- *Build long-term and trusting relationships*. Sustainable collaboration requires time to establish mutual trust. Create a safe and comfortable setting where participants can meaningfully shape the design process. Especially in early stages, consider including trusted stakeholders (eg, family members or support workers) to facilitate familiarity and confidence.
- *Address power dynamics and clarify roles*. Discuss expectations, roles, and decision-making responsibilities early and explicitly. Move beyond consultation by showing participants how their input has influenced decisions. Continuous reflection and transparent communication strengthen mutual respect and shared accountability.
- *Use methods close to lived experience*. Apply participatory methods that connect to everyday contexts, such as contextual interviews, observations with discussion, cognitive walk-throughs, or think-aloud sessions. Conducting co-creation activities in familiar environments ensures that insights reflect participants’ real-life experiences and capacities.
- *Monitor group dynamics*. Be attentive to how participants experience group settings. Some individuals with MID or ASD may find large groups overwhelming. Adjust group size and facilitation to participants’ comfort levels and communication styles.

### Conclusions

Reflection on an inclusive research and design process revealed that attention must be paid to tensions between project efficiency and meaningful inclusion, underlining the importance of ongoing coordination of the design process, collaboration, and flexibility in transdisciplinary settings. A stepwise approach, such as design thinking, proved helpful in structuring the process, continuous evaluation, and reducing these tensions. Inclusive methods, such as preparatory work, clear roles, accessible materials, and iterative feedback, supported active engagement throughout the project. These methods led to a shift in ownership, allowing co-researchers to gain more influence and actively shape both the development process and the content of the technology. These findings extend understanding of how inclusive design methods facilitate development processes and provide insights into how to enhance equity and relevance in inclusive technology design for individuals with complex care needs, such as individuals with MID or ASD.

## Supplementary material

10.2196/81941 Multimedia Appendix 1 Interview guide and questionnaire Work Package.

## Abbreviations

ASD: autism spectrum disorder
CeHRes: Center for eHealth Research
MID: mild intellectual disability
NASSS: Non-adoption, Abandonment, Scale-up, Spread, and Sustainability
SVA: sensitive virtual assistant
ViP: Vision in Product Design
WP: work package
