# Supplementary material for: Reflections on the Inclusive Co-Design Process of a Virtual Assistant for Individuals With Complex Care Needs: Mixed Methods Study
Source: JMIR Hum Factors. 2026 Feb 25;13:e81941. doi: 10.2196/81941 (PMC12935421; doi:10.2196/81941)
Supplement: Multimedia Appendix 1 [file humanfactors-v13-e81941-s001.docx]

Interview guide and questionnaire Work Package 1 co-researchers

**Introduction:**

Welcome to this interview, thank you for participating. We will be discussing the project in which we are collaboratively and inclusively developing and implementing a chatbot for vulnerable care recipients. I’m particularly interested in your personal experiences during this past work package.

As stated in the information letter, this interview will be recorded so that I can review it later for research purposes. The recording will be stored securely and will only be accessible to the researchers. The interview will last approximately one hour, during which we will go through and complete a questionnaire together. If you have any questions or wish to stop at any time, please let me know.

**Background information**

1. What is your role/position in de project?

**Evaluation of activities:**

*Focus groups with care recipients*

1. Preparation: Drafting protocol, recruitment & pilot
2. Execution: Conducting focus groups with care recipients
3. Processing: Evaluating focus groups with care recipients

*Focus groups with care providers*

1. Preparation: Drafting protocol and recruitment
2. Execution: Conducting focus groups with care providers
3. Processing: Evaluating focus groups with care providers

*Establishing interest profiles*

1. Formulating themes based on data from focus groups
2. Translating themes into interests

*Setting up a sounding board group*

1. Preparation: Recruiting participants and drafting the agenda
2. Execution: Holding the sounding board group
3. Processing: Evaluating the sounding board group

*Literaturstudy*

1. Collecting literature
2. Analazing literature
3. What was your role in the *activity*?
4. What tasks were involved?
5. Were the tasks related to the *activity* clear to you? Please give a score.

Totally not clear Very clear

|  |  |  |  |  |  |  |  |  |
| --- | --- | --- | --- | --- | --- | --- | --- | --- |

1 2 3 4 5 6 7 8 9 10

1. Why do you think that?
2. Who did you work with on these tasks?
3. How did you experience the collaboration during this task?


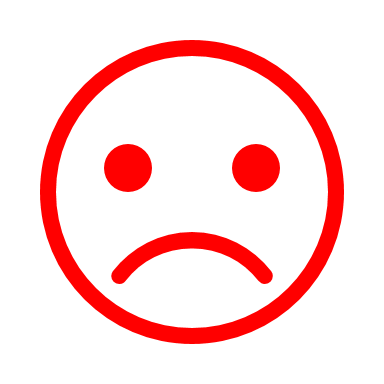

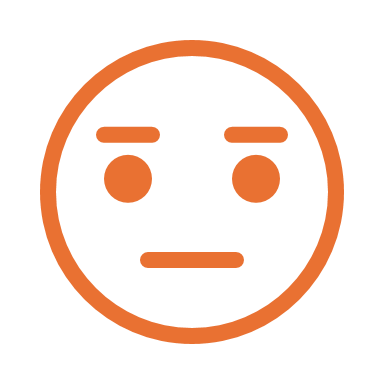

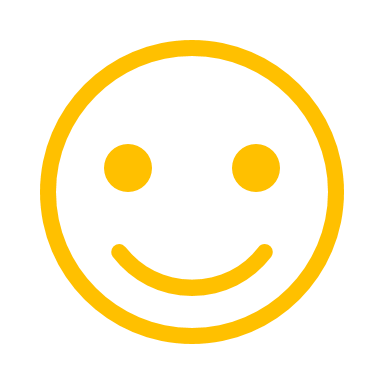

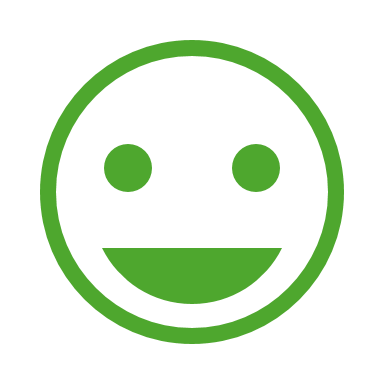


Very good good not so good bad

- 1. What went well and what could have been improved?

1. Are there any activities missing?


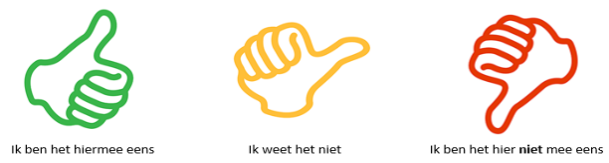

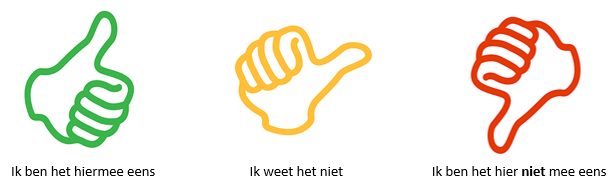


yes no

1. If so, which activity is still missing?

**Evaluatie project algemeen:**

1. What were the goals of this work package?
2. Have these goals been achieved?


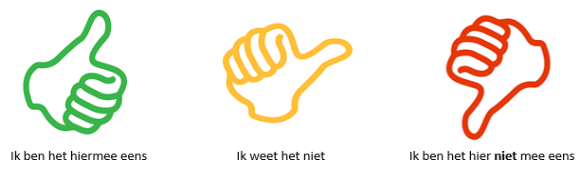


yes partially no

1. Why do you think that?
2. How many stars would you give your own contribution to the goals of this work package?


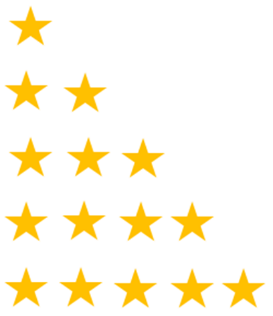


1. Why did you give that number of stars?

**Personal evaluations**

1. Did you learn anything from collaborating as a citizen scientist on this work package?


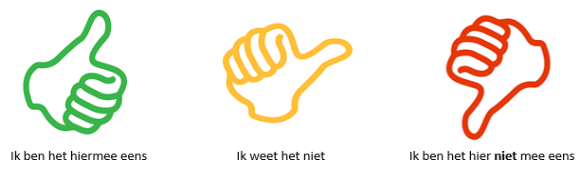


yes partially no

1. If so, what did you learn?
2. What went well in the collaboration during this work package?
3. What could have been improved?
4. How will you take this forward into the next phase of the project?
5. How many stars would you give the collaboration between citizen scientists within this work package?


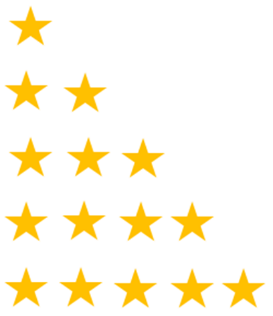


1. Why did you give that number of stars?
2. How do you feel about participating in this project? Please give a score.

Totally not nice Very nice

|  |  |  |  |  |  |  |  |  |
| --- | --- | --- | --- | --- | --- | --- | --- | --- |

1 2 3 4 5 6 7 8 9 10

1. Why do you give this score?

**Closing**

1. How did you experience completing this questionnaire together?
2. Do you have any questions or anything else you’d like to share?
